# First Report on the use of Loop-X Image Acquisition Technology for Sacral Neuromodulation

**DOI:** 10.1590/S1677-5538.IBJU.2025.9913

**Published:** 2025-06-20

**Authors:** Marcio Augusto Averbeck, Cezar Hinnah, Fabiano Faschel de Freitas, Antonio Carlos Matos da Silva, Andrey Kowalski

**Affiliations:** 1 Unidade de Videourodinâmica, Hospital Moinhos de Vento Chefe de Neuro-Urologia Porto Alegre RS Brasil Chefe de Neuro-Urologia, Unidade de Videourodinâmica, Hospital Moinhos de Vento. Porto Alegre, RS, Brasil;; 2 Medtronic Minneapolis USA Medtronic, Minneapolis, USA;; 3 Brainlab Robotic Suite Munich Germany Brainlab Robotic Suite, Munich, Germany

To the editor,

We are pleased to report the first global clinical application of the Loop-X intraoperative image acquisition system to guide sacral neuromodulation (SNM) implantation for refractory overactive bladder (OAB), carried out in Jan 2025 at Moinhos de Vento Hospital in Porto Alegre, Brazil.

As a well-established third-line treatment for OAB, SNM involves the strategic implantation of an electrode adjacent to the third sacral nerve root (S3), which plays a pivotal role in modulating bladder and urethral sphincter function (1, 2). The standard two-stage procedure includes an initial test phase lasting 1 to 2 weeks, during which patient response is evaluated using baseline bladder diaries as reference. In patients who demonstrate significant improvement, a permanent implantable pulse generator is subsequently placed to deliver chronic sacral nerve stimulation, with proven long-term benefits to quality of life.

**Figure 1:**
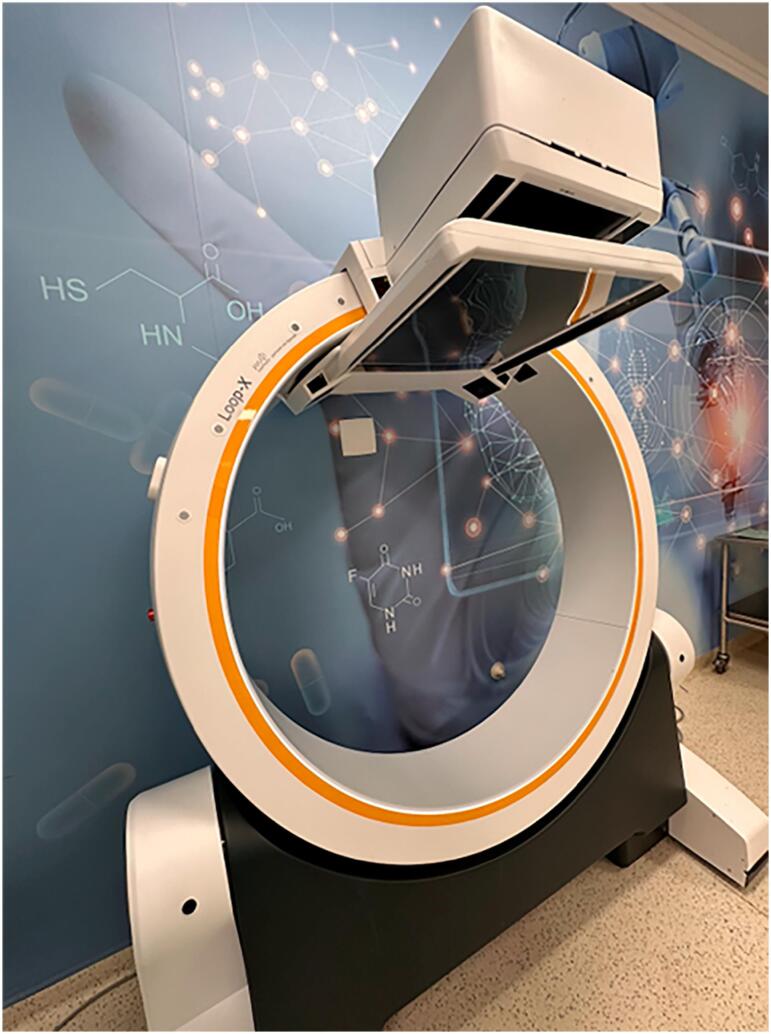
Loop-X intraoperative image acquisition technology.

**Figure 2:**
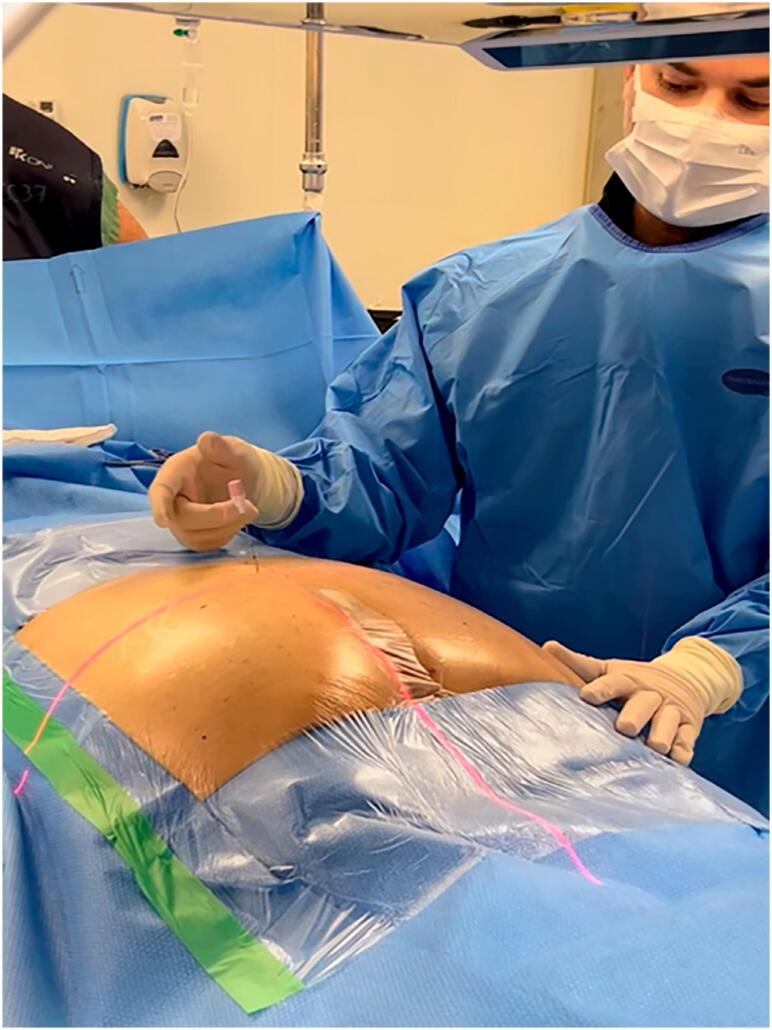
Laser marks guiding the exact point of needle entry.

**Figure 3:**
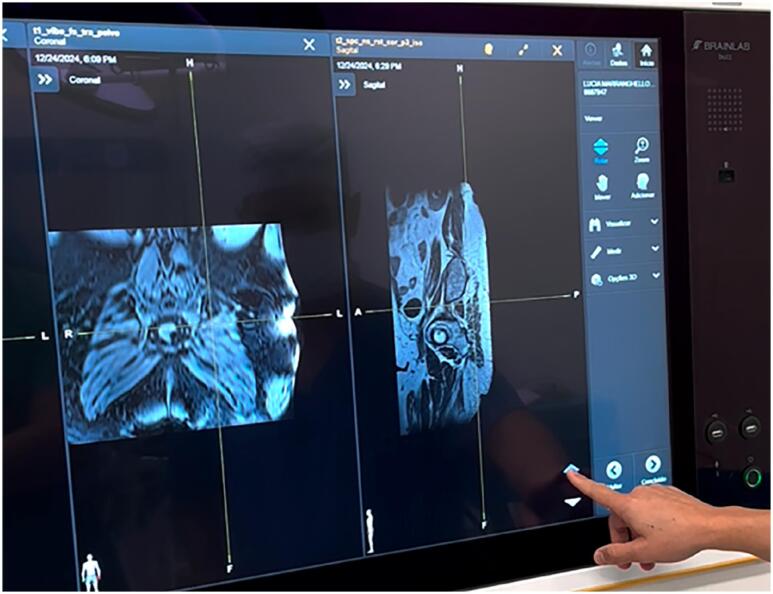
MRI Image fusion.

**Figure 4:**
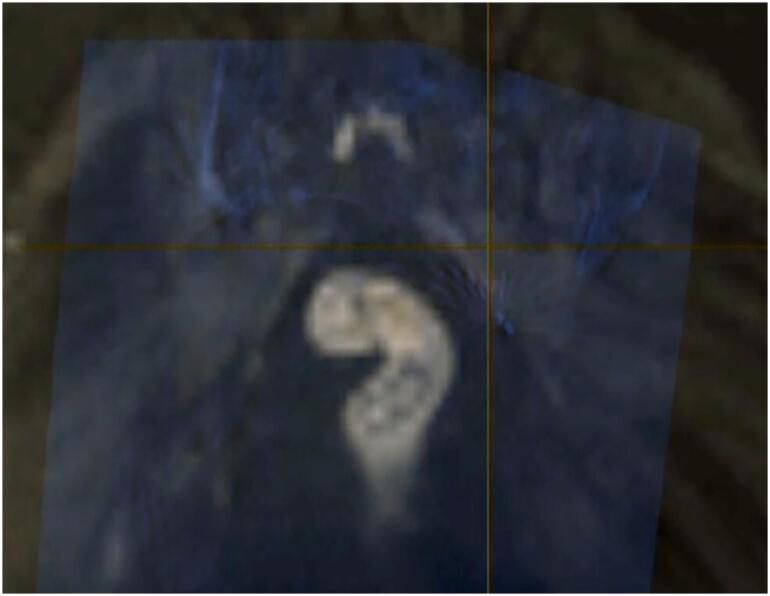
Electrode poles 0 and 1 demonstrated alongside the third sacral nerve (S3) in the coronal reconstruction (MRI image fusion).

The Loop-X system represents a novel advancement in intraoperative imaging, providing both 2D and 3D visualization capabilities (Figure-1). It enables the real-time fusion of high-definition preoperative magnetic resonance imaging with intraoperative fluoroscopy (Figures 2 to 4). This integration allows for unparalleled precision in targeting sacral nerve roots, reducing procedure time and enhancing safety and accuracy. At Moinhos de Vento Hospital, we successfully employed Loop-X technology to guide the implantation of a SureScan MRI-compatible lead (Medtronic, Minneapolis, USA). This marks the first known use of this robotic imaging platform for SNM worldwide.

Loop-X is one of several technologies comprising the Brainlab Robotic Suite, designed to support high-precision, image-guided surgical interventions. The system incorporates intraoperative 3D imaging, robotic guidance, and full digital integration. Its high degree of automation ensures procedural consistency and optimized patient outcomes.

We believe this case sets a precedent for the integration of advanced robotics and imaging technologies in functional urology. We encourage further reporting and discussion on the use of such platforms to enhance precision and outcomes in SNM and beyond.

The Authors
